# Prevalence, awareness, treatment and control of hypertension, diabetes and hypercholesterolaemia among adults in Dande municipality, Angola

**DOI:** 10.5830/CVJA-2017-047

**Published:** 2018

**Authors:** M Pedro João, Brito Miguel, M Pedro João, Barros Henrique, Brito Miguel, Barros Henrique

**Affiliations:** CISA, Centro de Investigação em Saúde de Angola, Caxito, Angola; CISA, Centro de Investigação em Saúde de Angola, Caxito, Angola; EPIUnit, Instituto de Saúde Pública, Universidade do Porto, Porto, Portugal; EPIUnit, Instituto de Saúde Pública, Universidade do Porto, Porto, Portugal; Escola Superior de Tecnologia da Saúde de Lisboa Instituto Politécnico de Lisboa, Lisboa, Portugal; Faculdade de Medicina, Universidade do Porto, Porto, Portugal

**Keywords:** epidemiology, hypertension, diabetes, hypercholesterolaemia, ub-Saharan Africa

## Abstract

**Objectives:**

To estimate the prevalence, awareness, treatment and control of hypertension, diabetes and hypercholesterolaemia in an Angolan population aged 15 to 64 years and to determine relationships with sociodemographic, behavioural and anthropometric characteristics.

**Methods:**

A total of 2 354 individuals were assessed for behavioural, sociodemographic and physical characteristics in a cross–sectional, community–based survey. Post–stratification survey weights were applied to obtain prevalence levels. Adjusted odds ratios for each variable related to the conditions were calculated using logistic regression models.

**Results:**

Overall, the prevalence of hypertension was 18.0%, diabetes 9.2% and hypercholesterolaemia 4.0%. Among hypertensive individuals, the awareness rate was 48.5%; 15.8% were on treatment and 9.1% had their blood pressure controlled. Only 10.8% were aware they had diabetes, 4.5% were on treatment and 2.7% were controlled. The awareness level for hypercholesterolaemia was 4.2%, with 1.4% individuals on treatment and 1.4% controlled.

**Conclusions:**

The prevalence levels of hypertension and diabetes, which were higher than previous findings for the region, together with the observed low rates of awareness, treatment and control of all conditions studied, constitute an additional challenge to the regional health structures, which must rapidly adapt to the epidemiological shift occurring in this population.

Cardiovascular disease (CVD), a major cause of non-communicable diseases (NCDs), was responsible for 17.5 million deaths worldwide in 2012, most occurring in low- and middle-income countries (LMIC). In Africa, the frequency of NCDs is rising rapidly, reflecting the combined effect of population growth andageing, as well as nutritional and epidemiological transitions.^1^

A large proportion of CVD is the result of exposure to modifiable risk factors (tobacco and alcohol consumption, unhealthy diet and physical inactivity), which influence metabolic pathways and ultimately result in obesity, hypertension, diabetes or hypercholesterolaemia.^1^,^2^ Together, these known adverse conditions explain approximately half of CVD cases, as demonstrated in the MONICA project and the INTERHEART study.^3^,^4^

Among the African population participating in the INTERHEART study, five risk factors (smoking, diabetes, hypertension, abdominal obesity and an elevated apolipoprotein B to apolipoprotein A-1 ratio) accounted for 89.2% of the population-attributable risk for the first myocardial infarction.5 The same study suggested that uncontrolled major risk factors have a larger impact on the burden of CVD in Africa than elsewhere in the world.^5^

If the current trends persist, the risk of dying from NCDs will increase in the African region. However, this rising risk could be reversed by reaching the proposed targets for six behavioural and physiological risk factors (tobacco and alcohol use, salt intake, obesity and increased blood pressure and glucose levels) out of the nine global targets proposed by the World Health Organisation (WHO) in the Global Action Plan for the Prevention and Control of NCD 2013–2020.^6^,^7^

To follow the achievement of those goals, there is a need for sound and updated epidemiological data from all regions of the world. The majority of published studies for the African region are conducted at hospital services, which does not allow one to detect risk factors, awareness rates and prevalence of such conditions in the general population.^8^-^10^ To provide core data on established risk factors for the major NCDs within the context of low-resource settings, WHO designed the STEPwise approach to Surveillance (STEPS).^11^ STEPS uses a modular structure with standardised questions and protocols, allowing adjustment of its application and appropriate comparisons across surveys.^11^

In Angola, infectious disease and maternal and child health-related problems remain the major causes of morbidity and mortality.^12^ However, an increased burden of NCDs has been observed, particularly CVD, which was responsible for 9% of adult deaths in 2013.^13^ Beyond general vital statistics, specific epidemiological information on CVD risk factors in Angola is based on only four local studies published after 2000: a survey of 667 adult students of Health Sciences in Lubango (prevalence of hypertension of 23.5%),^14^ a study conducted among 615 active employees of the University Agostinho Neto, Luanda (prevalence of hypertension 45.2% and hypercholesterolaemia 11.1%),^15^ 1 464 participants surveyed in the Dande Health and Demographic Surveillance System (Dande-HDSS) catchment area (23% prevalence of hypertension),^16^ and a study of 421 subjects from a rural community of Angola (2.8% prevalence of diabetes).^17^

Building on the work carried out by Pires and colleagues,^16^ and based on the STEPS methodology,^11^ this study aimed to expand the sample population to the 15- to 24-year-old group, and to estimate the prevalence, awareness, treatment and control of hypertension, diabetes and hypercholesterolaemia, and its association with sociodemographic (gender, age, education and area of residence), behavioural (alcohol and tobacco consumption) and anthropometric [body mass index (BMI) and abdominal obesity] variables among 15- to 64-year-olds in the Dande-HDSS population.

## Methods

A cross-sectional, community-based survey was conducted from September 2013 to March 2014 in the catchment area of the Dande-HDSS, located in Dande municipality of Bengo Province, Angola.^18^ A representative gender- and age-stratified random sample list of 3 515 individuals, aged between 15 and 64 years, was drawn, as described previously.^19^ Of these, we were able to examine 2 484 (70.7%) individuals, 750 (21.3%) were unreachable and 281 (8.0%) refused to participate, thus approaching the predicted non-participation rate of 30%.^19^

For analysis, we excluded participants with missing anthropometric values (n = 14) and pregnant women (n =116) due to the fact that anthropometric parameters vary during pregnancy. Therefore 2 354 individuals (67.0%) were included in the final analysis.

Information on age, completed years of school education, alcohol and tobacco consumption, and the previous measurement of any of the conditions under investigation, were collected through a structured interview conducted by trained interviewers, following a previously published protocol for data collection based on the WHO STEPS manual version 3.0.^11^,^19^

For this analysis, age was categorised into five 10-year age groups: 15 to 24, 25 to 34, 35 to 44, 45 to 54 and 55 to 64 years old. Education was categorised according to the number of completed years of schooling: none, one to four years, five to nine years, and 10 years or more. Area of residence was classified as rural or urban, as previously described.^18^ Alcohol consumption was defined as none if participants reported no alcohol consumption; occasional if participants reported drinking alcohol two or less days per week; and frequent if drinking any alcohol three or more days per week. Current tobacco smokers were defined as participants who reported smoking at least one cigarette per day.

Previous measurements of blood pressure, and glucose orcholesterol levels in the last year were requested from all participants. In the case of a positive answer, participants were questioned about their awareness of a previous diagnosisof hypertension, diabetes or hypercholesterolaemia made bya healthcare worker. Any individual was considered undertreatment if he/she indicated the use of a specific medication;a participant was considered controlled if they had a currentnormal value.

Certified health professionals conducted all anthropometric and clinical measurements, as described previously.^19^Anthropometric measurements were performed with individualswearing light clothing and no footwear, and an overnight fastwas requested of all participants.

Body mass and height were measured using a digital scaleSECA 803 (SECA United Kingdom, Birmingham, UK) and a portable stadiometer SECA 213 (SECA United Kingdom,Birmingham, UK). BMI was defined as the body mass (kg) divided by the square of the body height (m^2^), and furthercategorised according to WHO as underweight (< 18.5 kg/m^2^),normal (18.5 to 24.99 kg/m^2^), overweight (25.0 to 29.99 kg/m^2^)and obese (≥ 30 kg/m^2^).^20^

Waist and hip circumferences were measured usingcircumference tape SECA 203 (SECA United Kingdom,Birmingham, UK). The waist-to-hip ratio was calculated as thecircumference of the waist (cm) to that of the hips (cm), andabdominal obesity was defined as waist-to-hip ratio ≥ 0.9 formen and ≥ 0.85 for women.^21^

Blood pressure was measured on the right arm with the automatic sphygmomanometer OMRON M6 Comfort (OMRONHealthcare Europe BV, Hoofddorp, The Netherlands), with theindividual seated, and using an appropriate cuff size. Three readings were done at three-minute intervals. The mean value of the last two measurements was used to determine the bloodpressure. Hypertension was defined as systolic blood pressure of≥ 140 mmHg and/or diastolic blood pressure ≥ 90 mmHg and/or use of antihypertensive drugs during the previous two weeks.^22^

Blood sugar was measured using a blood glucose meter ACCU-CHEK Aviva (Roche Diagnostic, Indianapolis, IN, USA) with ACCU-CHEK Aviva glucose reactive strips (Roche Diagnostic, Indianapolis, IN, USA). The definition of diabetes followed WHO diagnostic criteria of 126 mg/dl (6.9 mmol/l) glucose in a fasting blood sample,^23^ and/or use of antidiabetic drugs during the previous two weeks.

Total cholesterol in the blood was measured using a point-of-care device ACCUTREND Plus (Roche Diagnostic, Indianapolis, IN, USA) with ACCUTREND cholesterol reactive strips (Roche Diagnostic, Indianapolis, IN, USA). Hypercholesterolaemia was defined according to WHO diagnostic criteria for STEPS, with cholesterol ≥ 240 mg/dl (6.2 mmol/l) in a fasting blood sample,^2^,^11^, and/or use of anticholesterol drugs during the previous two weeks.

All procedures performed in this study were in accordance with the standards of the ethics committee of the Angolan Ministry of Health and with the 1964 Helsinki declaration and its later amendments. Written informed consent was obtained from all individual participants included in the study (in the case of those under 18 years old, from their parent or legal guardian). A copy of the signed consent form, as well as instructions regarding the fasting period and contact information, were delivered to each participant.

## Statistical analysis

Data were double entered into a PostgreSQL® database and SPSS® version 22 (IBM Corp, Armonk, NY, USA) was used for statistical analysis. Post-stratification survey weights were calculated using the known gender and categorical age distribution of the Dande-HDSS population,^17^ and these were used in all further calculations. Descriptive data are reported as absolute frequencies and percentages or means and standard deviations (SD), as appropriate.

To facilitate comparisons with other studies, the prevalence of the three conditions under study was determined for three age groups: 15 to 64, 18 to 64 and 25 to 64 years. Logistic regression models were fitted to the categorical variable of age because of its known effect on hypertension, diabetes and hypercholesterolaemia. Gender-specific adjusted odds ratios (OR) were estimated for each variable (age, residence, education, BMI, abdominal obesity, tobacco and alcohol consumption) related to the conditions studied. A 95% confidence interval (95% CI) and a significance level of p < 0.05 were set for all applicable determinations.

## Results

The mean age of this population was 32.5 years (SD 13.6) with 63.0% (n = 1 482) women and the majority (81.0%) living in urban settings. Nearly 10% had never received any formal education, with men having completed more school years. Overall, almost a quarter of participants had abdominal obesity (36.5% of women and 12.4% of men), 6.8% were obese (10.6% of women and 2.8% of men), 6.2% were smokers (2.7% of women and 10.0% of men) and approximately two-fifths consumed alcohol occasionally or frequently, with a higher proportion of frequent drinkers among men (24.6 vs 10.9%) (Table 1).

**Table 1:** Socio-demographic, anthropometric and behavioural characteristics of the population (Caxito, 2016)

	All participants (n = 2 354)	Female (n = 1 222)	Male (n = 1 132)
Age (years) (n = 2 354)	% (95% CI)*	% (95% CI)*	% (95% CI)*
15–24	36.2 (34.3–38.1)	30.1 (27.6–32.7)	42.7 (39.9–45.6)
25–34	25.9 (24.2–27.7)	25.4 (23.0–27.9)	26.5 (24.0–29.1)
35–45	16.1 (14.7–17.6)	18.7 (16.6–20.9)	13.3 (11.5–15.4)
45–54	12.6 (11.3–14.0)	15.3 (13.4–17.4)	9.7 (8.1–11.6)
55–64	9.2 (8.1–10.4)	10.6 (9.0–12.4)	7.8 (6.3–9.5)
Residence (n = 2 354)			
Urban	81.0 (79.4–82.5)	81.2 (78.9–83.3)	80.8 (78.4–83.0)
Rural	19.0 (17.5–20.6)	18.8 (16.7–21.1)	19.2 (17.0–21.6)
Education (years completed) (n = 2 348)			
None	9.3 (8.2–10.5)	16.6 (14.6–18.8)	1.4 (0.9–2.3)
1–4	23.1 (21.5–24.9)	34.5 (31.9–37.2)	10.9 (9.2–12.8)
5–9	42.2 (40.2–44.2)	35.7 (33.1–38.5)	49.2 (46.3–52.1)
> 10	25.4 (23.7–27.2)	13.1 (11.4–15.2)	38.5 (35.7–41.4)
BMI class (kg/m^2^) (n = 2 354)			
Underweight (< 18.5)	11.3 (10.1–12.6)	10.2 (8.7–12.1)	12.5 (10.7–14.5)
Normal (18.5–24.9)	66.1 (64.1–67.9)	58.7 (55.9–61.4)	74.0 (71.4–76.5)
Overweight (25.0–29.9)	15.8 (14.4–17.3)	20.5 (18.4–22.9)	10.7 (9.0–12.6)
Obese (≥ 30)	6.8 (5.9–7.9)	10.6 (9.0–12.4)	2.8 (2.0–4.0)
Abdominal obesity (n = 2 354)			
No	75.1 (73.3–76.8)	63.5 (60.8–66.2)	87.6 (85.6–89.4)
Yes	24.9 (23.2–26.7)	36.5 (33.8–39.2)	12.4 (10.6–14.4)
Tobacco smoking (n = 2 342)			
Non-current	93.8 (92.7–94.7)	97.3 (96.2–98.1)	90.0 (88.1–91.6)
Current	6.2 (5.3–7.3)	2.7 (1.9–3.8)	10.0 (8.4–11.9)
Alcohol consumption (n = 2 335)			
No consumption	63.8 (61.8–65.7)	69.5 (66.9–72.0)	57.6 (54.7–60.4)
Occasional (< 3 days per week)	18.8 (17.2–20.4)	19.6 (17.5–21.9)	17.8 (15.7–20.2)
Frequent (≥ 3 days per week)	17.5 (16.0–19.1)	10.9 (9.2–12.7)	24.6 (22.2–27.2)

^*^Post-stratification weights used as described in the methods section.

The prevalence of hypertension in the general population was 18.0%, reaching 20.0% in those over 18 years of age, and 26.6% in those aged 25 to 64 years (Table 2). This prevalence was always higher among women than men, but with no statistically significant relationship (data not shown).

**Table 2:** Prevalence of hypertension, diabetes and hypercholesterolaemia by gender and age (Caxito, 2016)

	*All Participants*			*Female*			*Male*		
	*15–64 years (n = 2 354)*	*18–64 years (n = 2 100)*	*25–64 years (n = 1 503)*	*15–64 years (n = 1 222)*	*18–64 years (n = 1 116)*	*25–64 years (n = 854)*	*15–64 years (n = 1 132)*	*18–64 years (n = 984)*	*25–64 years (n = 649)*
Hypertension, % (95% CI)	18.0 (16.5–19.6)	20.0 (18.4–21.8)	26.6 (24.4–28.9)	20.0 (17.8–22.3)	21.8 (19.5–24.3)	27.8 (24.9–30.8)	15.9 (13.9–18.1)	18.1 (15.8–20.6)	25.1 (21.9–28.6)
Diabetes, % (95% CI)	9.2 (8.1–10.4)	9.8 (8.6–11.2)	11.9 (10.3–13.6)	8.9 (7.4–10.6)	9.3 (7.8–11.2)	10.8 (8.9–13.0)	9.6 (8.0–11.4)	10.4 (8.7–12.5)	13.5 (11.0–16.3)
Hypercholesterolaemia, % (95% CI)	4.0 (3.2–5.0)	4.4 (3.5–5.5)	5.5 (4.4–6.9)	5.6 (4.3–7.2)	6.0 (4.7–7.8)	7.4 (5.7–9.5)	2.0 (1.2–3.2)	2.4 (1.5–3.8)	2.9 (1.8–4.8)

The overall prevalence of diabetes among participants aged 15 to 64 years was 9.2%; the prevalence among those over 18 years old was 9.8%, and 11.9% in those aged over 25 years (Table 2). Men had a higher OR than women for diabetes of 1.4 (95% CI: 1.0–1.8, data not shown).

Similar to that of hypertension and diabetes, the prevalence of hypercholesterolaemia was higher in the older age groups, with an estimated 5.5% in participants aged 25 to 64 years, and a lower prevalence of 4.0% in the overall population (Table 2). Women had an OR of 2.3 (95% CI: 1.3–4.0, data not show) for hypercholesterolaemia.

Only five participants (0.2%; 95% CI: 0.1–0.4, data not shown) presented all three conditions, but 22.0% (95% CI: 18.4–26.2, data not shown) of hypertensive participants had an associated condition, as did 37.2% (95% CI: 31.1–43.7, data not shown) of participants with diabetes and 47.9% (95% CI: 36.7–59.3, data not shown) of those with hypercholesterolaemia. The most common associations were hypertension and diabetes, present in 71 individuals (3.0%; 95% CI: 2.4–3.7, data not shown).

The prevalence of hypertension was higher in rural areas (26.9 vs 15.9% in urban areas) for both genders. Individuals with lower levels of education had a higher prevalence of hypertension, with women with no formal education presenting an OR for hypertension of 4.3 (Table 3).

**Table 3:** Prevalence of hypertension and relation with other factors by gender (Caxito, 2016)

	*All Participants(n = 2 354)*	*Female (n = 1 222)*		*Male (n = 1 132)*	
*Associated factor*	Prevalence % (95% CI)*	Prevalence % (95% CI)*	Adjusted OR^^a, b^^ (95% CI)*	Prevalence % (95% CI)*	Adjusted OR^^a, b^^ (95% CI)*
Total	18.0 (16.5–19.6)	20.0 (17.8–22.3)	–	15.9 (13.9–18.1)	–
Age (years)					
15–24	2.8 (1.9–4.2)	1.9 (0.9–3.9)	1	3.5 (2.2–5.6)	1
25–34	12.3 (9.9–15.2)	10.6 (7.7–14.6)	6.6 (2.8–15.4)	14.3 (10.8–18.8)	4.6 (2.6–8.2)
35–44	25.6 (21.5–72.0)	26.8 (21.4–32.9)	20.3 (8.9–46.5)	23.8 (17.7-31.2)	8.7 (4.7-16.0)
45–54	38.7 (33.4–44.4)	39.6 (32.8–39.6)	36.6 (16.0–83.8)	37.3 (28.8–46.6)	16.2 (8.7–30.0)
55–64	51.6 (45.0–58.2)	53.5 (44.9–61.9)	63.4 (27.1–147.9)	48.9 (38.7–59.1)	26.4 (13.9–50.0)
Residence					
Urban	15.9 (14.3–17.6)	17.6 (15.3–20.1)	–	14.0 (11.9–16.4)	–
Rural	26.9 (23.0–31.2)	30.0 (24.4–36.2)	–	23.5 (18.4–29.6)	–
Education (years completed)					
None	45.4 (38.9–52.0)	45.5 (38.8–52.4)	4.3 (1.8–10.2)	46.7 (24.8–69.9)	2.0 (0.6–6.5)
1–4	24.9 (21.4–28.7)	23.3 (19.5–27.6)	2.4 (1.0–5.4)	29.8 (22.5–38.4)	0.8 (0.5–1.5)
5–9	12.7 (10.8–14.9)	10.3 (7.8–13.6)	2.2 (0.9–5.1)	14.5 (11.8–17.7)	0.9 (0.6–1.4)
> 10	10.4 (8.2–13.1)	4.4 (2.1–8.8)	1	12.6 (9.8–16.1)	1
BMI class (kg/m^2^)					
Underweight (< 18.5)	11.0 (7.8-15.3)	12.9 (8.1-19.0)	1	9.3 (5.5-15.2)	1
Normal (18.5–24.9)	15.2 (13.5–17.1)	17.0 (14.4–19.9)	1.1 (0.6–2.1)	13.7 (11.5–16.2)	1.3 (0.7–2.5)
Overweight (25.0–29.9)	25.8 (21.6–30.5)	23.9 (19.0–29.5)	1.2 (0.6–2.3)	29.2 (21.8–37.8)	2.2 (1.1–4.7)
Obese (≥ 30)	37.3 (30.2–45.0)	34.9 (27.2–43.4)	2.0 (1.0–4.1)	48.5 (32.5–64.8)	5.1 (1.9–13.4)
Abdominal obesity					
No	12.1 (10.6–13.7)	12.6 (10.5–15.2)	1	11.6 (9.7–13.7)	1
Yes	35.7 (31.9–39.6)	32.5 (28.3–37.0)	1.6 (1.2-2.3)	45.7 (37.7–54.0)	2.8 (1.8–4.3)
Tobacco smoking					
Non-current	17.3 (15.8–18.9)	18.9 (16.7–21.2)	–	15.5 (13.4–17.8)	–
Current	26.7 (20.2–34.4)	50.0 (34.1–65.9)	–	20.4 (14.0–28.7)	–
Alcohol consumption					
No consumption	14.2 (12.6–16.1)	18.1 (15.7–20.9)	1	9.1 (7.2–11.6)	1
Occasional (< 3 days per week)	23.5 (19.8–23.5)	21.4 (16.7–27.1)	0.9 (0.6–1.4)	26.0 (20.4–32.5)	2.5 (1.6–4.0)
Frequent (≥ 3 days per week)	25.5 (21.5–25.5)	28.0 (21.1–36.2)	1.7 (1.1–2.7)	24.3 (19.6–29.7)	2.5 (1.7–3.9)

*Post-stratification weights used as described in the methods section; ^a^Adjusted for age (categorical: 15–23, 25–34, 35–44, 45–54, and 55–64); ^b^Only variables with relations with statistical significance shown.

Hypertension was higher among the obese (34.9% of women and 48.5% of men) and individuals with abdominal obesity (32.5% of women and 45.7% of men), with a higher OR in men for both conditions (Table 3). Hypertension prevalence was also higher among current smokers (50.0% in women and 20.4% in men) and frequent alcohol drinkers (28.0% in women and 24.3% in men). Men presented a higher OR for hypertension than women, related to the consumption of alcohol (Table 3).

Residents in urban areas presented a higher prevalence of diabetes, with a significantly higher OR for diabetes in men. Participants with lower education levels had a higher prevalence of diabetes, but without statistical significance (Table 4). With regard to anthropometric variables, there was a higher prevalence of diabetes among obese participants (17.1% in women and 24.2% in men) and those with abdominal obesity (8.8% in women and 24.3% in men). Men with obesity (2.4 vs underweight) and abdominal obesity (2.3 vs no abdominal obesity) presented higher ORs for diabetes than women (2.1 for obese vs underweight and 1.5 for abdominal obesity) (Table 4).

**Table 4:** Prevalence of diabetes and relation with other factors by gender (Caxito, 2016)

	*All Participants(n = 2 354)*	*Female (n = 1 220)*		*Male (n = 1 128)*	
*Associated factor*	Prevalenc % (95% CI)*	Prevalence % (95% CI)*	Adjusted ORM^^a, b^^ (95% CI)*	Prevalence % (95% CI)*	Adjusted OR^^a, b^^ (95% CI)*
Total	9.2 (8.1–10.4)	8.9 (7.4–10.6)	1	9.6 (8.0–11.4)	1.4 (1.0–1.8)
Age (years)					
15–24	4.4 (3.2–6.0)	4.4 (2.7–7.0)	1	4.4 (2.9–6.6)	1
25–34	5.6 (4.0–7.7)	3.2 (1.8–5.9)	0.8 (0.3–1.7)	8.0 (5.4–11.6)	1.9 (1.0–3.5)
35–44	13.2 (10.2–17.0)	12.7 (9.0–17.7)	3.3 (1.7–6.2)	13.9 (9.3–20.3)	3.4 (1.8–6.5)
45–54	19.3 (15.2–24.2)	17.6 (12.9–23.7)	4.8 (2.6–9.0)	22.2 (15.4–30.9)	6.2 (3.3–11.6)
55–64	17.2 (12.8–22.8)	15.5 (10.3–22.7)	4.0 (2.0–8.0)	20.7 (13.5–30.4)	5.6 (2.8–11.0)
Residence					
Urban	9.8 (8.5–11.2)	9.2 (7.5–11.1)	1.6 (0.9–2.8)	10.4 (8.6–12.6)	2.6 (1.4–4.9)
Rural	6.8 (4.8–9.5)	7.4 (4.7–11.6)	1	6.0 (3.6–10.1)	1
Education (years completed)					
None	11.5 (7.9–16.5)	11.9 (8.1-17.1)	–	6.7 (1.2-29.8)	–
1–4	11.7 (9.2–14.6)	10.0 (7.5-13.3)	–	17.2 (11.5-24.9)	–
5–9	8.3 (6.7–10.1)	7.1 (5.1–9.9)	–	9.0 (6.9–11.6)	–
> 10	7.7 (5.9–10.2) 7.7 (5.9–10.2)	6.2 (3.4–11.1)	–	8.3 (6.1–11.3)	–
BMI class (kg/m^2^)					
Underweight (< 18.5)	7.5 (4.9–11.4)	4.0 (1.7–9.0)	1	10.7 (6.6–16.9)	1
Normal (18.5–24.9)	7.8 (6.6–9.2)	7.7 (5.9–9.9)	2.0 (0.7–5.1)	7.9 (6.3–9.9)	0.7 (0.4–1.2)
Overweight (25.0–29.9)	12.4 (9.4–16.1)	10.4 (7.2–14.7)	2.4 (0.9–6.5)	16.5 (11.0–24.2)	1.1 (0.5–2.3)
Obese (≥ 30)	18.6 (13.4–25.4)	17.1 (11.5–24.5)	3.9 (1.4–11.1)	24.2 (12.8–41.0)	1.7 (0.6–4.5) 1.7 (0.6–4.5)
Abdominal obesity					
No	7.0 (5.9–8.3)	3.5 (2.3–5.2)	1	7.5 (6.0–9.3)	1
Yes	15.9 (13.1–19.0)	8.8 (6.4–12.2)	1.5 (1.0–2.3)	24.3 (17.9–32.0)	2.3 (1.4–3.8)
Tobacco smoking					
Non-current	8.8 (7.6–10.0)	8.6 (7.2–10.4)	–	8.9 (7.3–10.8)	–
Current	14.4 (9.6–21.0)	17.6 (8.3–33.5)	–	13.3 (8.2–20.8)	–
Alcohol consumption					
No consumption	8.9 (7.6–10.5)	8.7 (6.9–10.8)	–	9.2 (7.2–11.7)	–
Occasional (< 3 days per week)	10.5 (8.0–13.7)	10.1 (6.9–14.6)	–	11.0 (7.4–16.1)	–
Frequent (≥ 3 days per week)	8.8 (6.4–12.0)	8.3 (4.7–14.3)	–	9.1 (6.2–13.0)	–

*Post-stratification weights used as described in the methods section; ^a^Adjusted for age (categorical: 15–23, 25–34, 35–44, 45–54, and 55–64); ^b^Only variables with relations with statistical significance shown.

For current smokers and occasional consumers of alcohol the prevalence of diabetes was higher, but with no significant relationship (Table 4). No significant relationships were found with education, residence, BMI, abdominal obesity, tobacco smoking and alcohol consumption; however, the prevalence of hypercholesterolaemia was higher among less educated individuals, the obese, smokers and frequent alcohol drinkers (Table 5).

**Fig. 1:**
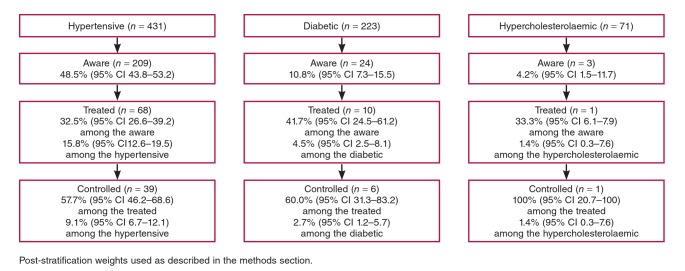
Frequencies, awareness, treatment and control of hypertension, diabetes and hypercholesterolaemia.

The majority of the population (61.5%; n = 1 460) reported previous measures of blood pressure, and nearly half (48.5%) of the hypertensive participants were aware of their condition. Only 32.5% of the aware hypertensive participants were on treatment and 57.7% of them had their blood pressure controlled. This represented only 9.1% of all hypertensive participants (Fig. 1).

**Table 5:** Prevalence of hypercholesterolaemia and relation with other factors by gender (Caxito, 2016)

	*All Participants(n = 2 354)*	*Female (n = 1 220)*		*Male (n = 1 128)*	
*Associated factor*	Prevalence % (95% CI)^*^	Prevalence % (95% CI)^*^	Adjusted OR^a, b^ (95% CI)*	Prevalence % (95% CI)^*^	Adjusted OR^a, b^ (95% CI)*
Total	4.0 (3.2–5.0)	5.6 (4.3–7.2)	2.3 (1.3–4.0)	2.0 (1.2–3.2)	1
Age (years)					
15–24	0.7 (0.3–1.8)	1.1 (0.4–3.2)	1	0.3 (0.1–1.9)	1
25–34	2.5 (1.4–4.3)	2.8 (1.4–5.7)	2.6 (0.6–10.8)	2.5 (1.1–5.3)	5.0 (0.8–31.6)
35–44	3.6 (2.0–6.4)	5.4 (2.9–9.6)	5.2 (1.4–20.0)	0.9 (0.2–4.7)	2.1 (0.2–24.4)
45–54	9.4 (6.3–13.7)	10.8 (6.9–16.7)	11.9 (3.30–42.7)	5.7 (2.5–12.8)	13.7 (2.1–88.1)
55–64	11.4 (7.6–16.8)	15.4 (10.0–23.0)	17.2 (4.8–61.9)	4.5 (1.6–12.5)	9.0 (1.2–69.5)
Residence					
Urban	3.9 (3.0–5.0)	5.6 (4.2–7.4)	–	1.8 (1.0–3.2)	–
Rural	4.2 (2.5–7.0)	5.3 (2.8–9.8)	–	3.5 (1.5–7.9)	–
Education (years completed)					
None	10.8 (7.0–16.2)	10.7 (6.9–16.3)	–	11.1 (2.0–43.5)	–
1–4	5.7 (3.9–8.3)	6.4 (4.3–9.5)	–	2.5 (0.7–8.8)	–
5–9	2.6 (1.7–4.1)	3.3 (1.9–5.9)	–	2.0 (1.0–3.9)	–
> 10	2.0 (1.0–3.7)	2.3 (0.8–6.5)	–	1.9 (0.9–4.0)	–
BMI class (kg/m^2^)					
Underweight (< 18.5)	2.3 (0.9–5.7)	3.2 (1.1–9.1)	–	1.2 (0.2–6.5)	–
Normal (18.5–24.9)	3.5 (2.6–4.7)	5.1 (3.6–7.3)	–	1.9 (1.1–3.3)	–
Overweight (25.0–29.9)	5.3 (3.3–8.3)	6.0 (3.6–10.1)	–	3.8 (1.5–9.3)	–
Obese (≥ 30)	6.7 (3.5–12.2)	8.6 (4.6–15.5)	–	–	–
Abdominal obesity					
No	2.4 (1.7–3.4)	3.5 (2.3–5.2)	–	1.5 (0.8–2.7)	–
Yes	8.1 (6.0–10.9)	8.8 (6.4–12.2)	–	5.9 (2.9–11.6)	–
Tobacco smoking					
Non-current	3.7 (2.9–4.8)	5.1 (3.9–6.7)	–	2.0 (1.2–3.3)	–
Current	6.4 (3.1–12.6)	17.9 (7.9–35.6)	–	2.5 (0.7–8.6)	–
Alcohol consumption					
No consumption	4.3 (3.2–5.6)	5.7 (4.2–7.7)	–	2.2 (1.2–4.1)	–
Occasional (< 3 days per week)	2.7 (1.4–5.0)	4.6 (2.5–8.6)	–	–	–
Frequent (≥ 3 days per week)	3.9 (2.2–6.7)	5.6 (2.6–11.6)	–	2.5 (1.1–5.7)	–

^*^Post-stratification weights used as described in the methods section; ^a^Adjusted for age (categorical: 15–23, 25–34, 35–44, 45–54, and 55–64); ^b^Only variables with relations with statistical significance shown; cNo cases in this category.

Only 7.3% (n = 172) of the population reported previous measurement of glycaemia, with a low awareness rate of 10.8% among participants with diabetes in this study. Of the aware participants, 41.7% were receiving treatment (4.5% of all hyperglycaemic participants) and 60.0% had a controlled blood sugar level (Fig. 1). Only 2.9% (n = 68) of participants reported previous measures of cholesterolaemia and only 4.2% of individuals with hypercholesterolaemia were aware of their condition (Fig. 1).

The hypertension awareness rate was higher among women (62.7%; 95% CI: 55.9–69.0) and older participants, without a difference regarding education level (Table 6). The diabetes awareness rate was higher among men (58.3%; 95% CI: 38.8– 75.5), older participants and those with higher education levels (Table 7). The hypercholesterolaemia awareness rate was higher among women (66.7%; 95% CI: 20.8–93.9), older age groups and higher education levels (Table 8). The treatment rate of all conditions was more prevalent in the older age groups and higher education levels, but the control rate was more frequent in younger participants.

Among the individuals who were aware of any of the three conditions, the advice most often given by healthcare professionals to follow non-pharmacological approaches for the management of cardiovascular risk factors was a change in dietary habits, with a decrease in salt and fat intake, and increased fruit and vegetable intake (Table 9).

**Table 6:** Awareness, treatment and control rates of hypertension by gender (Caxito, 2016)

	*Awareness*			*Treatment*			*Control*		
	All (n = 209) % (95% CI)	Female (n = 131) % (95% CI)	Male (n = 78) % (95% CI)	All (n = 68) % (95% CI)	Female (n = 41) % (95% CI)	Male (n = 27) % (95% CI)	All (n = 39) % (95% CI)	Female (n = 25) % (95% CI)	Male (n = 14) % (95% CI)
Education (years completed)									
None	21.5 (16.5–27.6)	34.4 (26.8–42.8)	0	17.6 (10.4–28.4)	26.8 (15.7–41.9)	3.7 (0.7–18.3)	10.3 (4.1–23.6)	16.0 (6.4–34.7)	0
1–4	31.1 (25.2–37.7)	40.5 (32.4–49.0)	15.4 (9.0–25.0)	27.9 (18.7–39.6)	39.0 (25.7–54.3)	11.1 (3.9–28.1)	25.6 (14.6–41.1)	40.0 (23.4–59.3)	0
5–9	28.2 (22.6–34.7)	22.1 (15.9–30.0)	38.5 (28.4–49.6)	29.4 (19.9–41.1)	26.8 (15.7–41.9)	33.3 (18.6–52.2)	33.3 (20.6–49.0)	36.0 (20.2–55.5)	28.6 (11.7–54.6)
> 10	19.1 (14.4–25.0)	3.1 (1.2–7.6)	46.2 (35.5–57.1)	25.0 (16.2–36.4)	7.3 (2.5–19.4)	51.9 (34.0–69.3)	30.8 (18.6–46.4)	8.0 (2.2–25.0)	71.4 (45.4–88.3)
Age (years)									
15–24	2.9 (1.3–6.1)	0.8 (0.1–4.2)	6.4 (2.8–14.1)	1.5 (0.3–7.9)	2.4 (0.4–12.6)	0	2.6 (0.5–13.2)	4.0 (0.7–19.5)	0
25–34	16.7 (12.3–22.4)	12.2 (7.7–18.9)	24.4 (16.2–34.9)	26.5 (17.4–38.0)	24.4 (13.8–39.3)	29.6 (15.9–48.5)	33.3 (20.6–49.0)	32.0 (17.2–51.6)	35.7 (16.3–61.2)
35–44	19.6 (14.8–25.5)	19.1 (13.3–26.7)	20.5 (13.0–30.8)	20.6 (12.7–31.6)	19.5 (10.2–34.0)	22.2 (10.6–40.8)	25.6 (14.6–41.1)	24.0 (11.5–43.4)	28.6 (11.7–54.6)
45–54	31.1 (25.2–37.7)	37.4 (29.6–45.9)	20.5 (13.0–30.8)	23.5 (15.0–34.9)	26.8 (15.7–41.9)	18.5 (8.2–36.7)	17.9 (9.0–32.7)	20.0 (8.9–39.1)	14.3 (4.0–39.9)
55–64	29.7 (23.9–36.2)	30.5 (23.3–38.9)	19.4 (19.4–39.0)	27.9 (18.7–39.6)	26.8 (15.7–41.9)	29.6 (15.9–48.5)	20.5 (10.8–35.5)	20.0 (8.9–39.1)	21.4 (7.6–47.6)

**Table 7:** Awareness, treatment and control rates of diabetes by gender (Caxito, 2016)

	*Awareness*			*Treatment*			*Control*		
	*All (n = 24) %*	*Female (n = 10) %*	*Male (n = 14) %*	*All >(n = 10) %*	*Female (n = 6) %*	*Male (n = 4) %*	*All (n = 6) %*	*Female (n = 5) >%*	*Male (n = 1) %*
Education (years completed)									
None	12.5	30.0	0.0	20.0	33.3	0	16.7	20.0	0
1–4	4.2	10.0	0.0	10.0	16.7	0	16.7	20.0	0
5–9	33.3	30.0	35.7	50.0	33.3	75.0	50.0	40.0	100.0
> 10	50.0	30.0	64.3	20.0	16.7	25.5	16.7	20.0	0
Age (years)									
15–24	8.3	20.0	0.0	20.0	33.3	0	33.3	40.0	0
25–34	12.5	10.0	14.3	10.0	16.7	0	16.7	20.0	0
35–44	20.8	10.0	28.6	20.0	16.7	25.5	16.7	20.0	0
45–54	25.0	20.0	28.6	10.0	16.7	0	0	0	0
55–64	33.3	40.0	28.6	40.0	16.7	75.0	33.3	20.0	100.0

**Table 8:** Awareness, treatment and control rates of hypercholesterolemia by gender (Caxito, 2016)

	*Awareness*			*Treatment*			*Control*		
	*All (n = 3) %*	*Female (n = 2) %*	*Male (n = 1) %*	*All (n = 1) %*	*Female (n = 1) %*	*Male (n = 0) %*	*All (n = 1) %*	*Female (n = 1) %*	*Male (n = 0) %*
Education (years completed)									
None	0	0	0	0	0	0	0	0	0
1–4	33.3	50.0	0	0	0	0	0	0	0
5–9	0	0	0	0	0	0	0	0	0
> 10	66.6	50.0	100.0	100.0	100.0	0	100.0	100.0	0
Age (years)									
15–24	0	0	0	0	0	0	0	0	0
25–34	0	0	0	0	0	0	0	0	0
35–44	33.3	50.0	0	100.0	100.0	0	100.0	100.0	0
45–54	66.6	50.0	100.0	0	0	0	0	0	0
55–64	0	0	0	0	0	0	0	0	0

**Table 9:** Non-pharmacological advice by health professionals to aware participants (Caxito, 2016)

	*Hypertension (n = 209)*	*Diabetes (n = 24)*	*Hypercholesterolaemia (n = 3)*
*Advice*	*% (95% CI)*	% (95% CI)*	% (95% CI)*
Reduce salt in your diet	78.5 (72.4–83.5)	100.0	100.0
Reduce fat in your diet	61.7 (55.0–68.0)	91.7	66.7
Eat at least five servings of fruit and/or vegetables each day	58.4 (51.6–64.8)	70.8	66.7
Reduce or stop alchool consumption	51.2 (44.5–57.9)	83.3	33.3
Start or do more physical activity	34.4 (28.3–41.1)	75.0	66.7
Quit using tobacco or don’t start	31.1 (25.2–37.7)	45.8	0
Maintain a healthy body weight or lose weight	30.1 (24.3-36.7)	75.0	66.7

^*^Due to the small sample size, the 95% CI was not determined.

## Discussion

The prevalence of hypertension among participants in the range of 15 to 64 years old was 18.0%. This value rose to 26.6% among participants aged 25 to 64 years, which is slightly higher than those previously described for Angola over the last eight years,^14^-^15^ particularly a study conducted in the same region in 2010,^16^ and the WHO age-standardised (25 to 64 years old) estimated hypertension prevalence for 2014 in Angola of 23.9% (95% CI: 16.3–31.1).^1^ More recently, a cross-sectional study conducted in Uganda, South Africa, Tanzania and Nigeria encountered an overall age-standardised prevalence of hypertension of 25.9%.^24^

The estimated 9.2% prevalence of diabetes (9.8% in urbanand 6.8% in rural areas) was higher than previous reports from Angola of 5.7% among an urban population (aged 20 to 72 years)in 2010,^15^ and 2.8% for a rural community (aged 30 to 69 years) in 2009.^17^ The value of 9.8% estimated in individuals older than 18 years is in the middle range of prevalence levels encountered in STEPS surveys, with values from 3.0% in Benin to 22.5% in Niger.^25^,^26^ This value also falls within the confidence intervals ofthe WHO estimate of 12.1% (95% CI: 5.6–18.9) for increasedblood glucose levels in those over 18 years in Angola for 2014.^1^

This rise in diabetes is aligned with the global tendency for this disease, which has increased faster in LMIC than in high-income countries since 1980.^27^ Since the end of the Angolancivil war in 2002, the population has been increasing and ageing.This, together with changes in food habits and the urbanisation process, may have led to the increased prevalence of diabetes in this region.

The prevalence of hypercholesterolaemia (5.3% among participants 25 and 64 years old) in this study was lower than that found in a previous study in Luanda among an older urban population.^15^ However, this value falls within a wide range of values from several STEPS surveys measuring the prevalence of total cholesterol, from 2.1% in Mozambique to 26.0% in Tanzania.^25^,^26^ This prevalence may also be tied to the ageing population and changes in dietary habits that most African countries are currently facing.^28^ There is a lack of solid knowledge regarding the prevalence levels of hypercholesterolaemia in Africa, mainly owing to the difficulties in determining values of blood cholesterol in African communities because of the high cost of laboratory tests. This situation presents a challenge when comparing research results.

As described in other studies worldwide, the clustering of risk factors helps to explain the known impacts of age, education and obesity on the occurrence of hypertension, diabetes and hypercholesterolaemia. The prevalence of these three conditions was higher among individuals with less education, and increased with age and BMI.

Obesity represents a major concern as a risk factor for CVD and NCDs in general, and is connected with the current nutritional transition in Africa, with a shift in the composition and structure of diets traditionally low in fat and high in unrefined carbohydrates toward higher intakes of refined carbohydrates, added sugars, fats and animal-source foods.^28^ This shift may have had an impact on the rise in incidence of diabetes over the past decades, revealed in recent literature reviews,^29^-^31^ as well as a WHO estimation of the rise in median prevalence of elevated total cholesterol for this region.^2^

Similar to this nutritional transition, the process of urbanisation underway in the region must be taken into consideration for future interventions. Living in an urban area has been associated with a two-fold increase in the prevalence of diabetes among this population, as described in other studies.^1^,^29^-^31^

Information regarding the awareness, treatment and control rates for the three conditions investigated is scarce for the African continent, except for hypertension; there are also some available data with regard to diabetes. Our findings for awareness of hypertension were higher than those calculated in 2010 for Africa, with an estimated 33.7% pooled awareness rate.^32^ Current values for awareness, treatment and control of hypertension are higher than in 2011 in the same population; results for awareness were 21.6% (95% CI: 17.0–26.9) in 2011 and 48.5% in the present study. Values for participants who were aware of their condition and on pharmacological treatment (13.9%, 95% CI: 5.9–29.1) increased to 32.5%; approximately one-third of participants were controlled in 2011 and more than half were controlled in our study. This may have resulted from the positive effect of identification of hypertensive individuals and medical follow up after the first survey in 2011.

Nonetheless, the levels of awareness about hypertensive status are still low, a situation common in Africa,^33^ with levels much lower than those in North America and Europe.^34^ A similar framework exists for diabetes awareness in Africa, with fewer than 50% of participants in one study aware of their condition.^29^ No data were found for awareness of total cholesterol levels.

The lack of primary healthcare facilities in this region, especially in rural areas, makes the low levels of previous measurements plausible. Furthermore, the current training of Angolan health professionals and the availability of clinical equipment are still focused on infectious diseases, not considering CVD a priority. Therefore initiatives promoting the awareness of CVD are lacking in the region, and proper monitoring of patients’ conditions does not occur.

Moreover, the information available to the population is not enough to convince patients to take lifelong medication in order to treat a condition, which is usually asymptomatic. Only one-third of participants with any of these conditions had access to treatment, which demonstrates the inadequacy of the region’s health system to help patients manage risk factors. Economic difficulties and the lack of drugs to address CVD may also help explain the low levels of treatment and control found.

Nevertheless, a positive note should be made as to the number of patients who had controlled levels of blood pressure, blood sugar and cholesterolaemia in this specific population. Considering that they were younger and better educated, they could have had easier access to drugs and health facilities. Also noteworthy, in the absence of access to drugs, physicians’ advice in most cases is to adopt non-pharmacological approaches to reducing modifiable risk factors, mainly associated with diet.

## Strengths and limitations of the study

Our study findings should be interpreted cautiously because the Dande-HDSS was developed as a district-level surveillance system in an urban and rural setting and is therefore not representative of the demographic structure of the country. In addition, age groups over 65 years old (known for higher rates of the conditions studied) were not considered owing to their low representation in the general structure of the population (3.6% of the Dande-HDSS population),^18^ which is a common practice for surveys conducted in sub-Saharan Africa.

Internal migration and the geographical isolation of some hamlets within the Dande-HDSS, together with the fact that working individuals were unavailable during the daytime,^17^ were reflected in the sampling definition, with a 30% non-participation rate. The distribution of non-respondents was uneven, with a higher proportion of younger people and men (data not shown). This may have caused instability in the estimates in some strata.

Participants were requested not to eat anything eight hours before participating in the study; however, it was difficult to measure adherence to this request, which adds uncertainty to the measures of blood glucose and cholesterol. We used dry chemistry devices to measure glycaemia and cholesterolaemia, but owing to high temperatures and humidity during field surveys, data collection was not possible in some cases, causing a higher number of missing data than expected.

Due to the many variables covered in the survey and to avoid drop-out of participants in future rounds, additional questions relating to awareness, pharmacological treatments and non-pharmacological approaches were conducted in a more detailed form in individual follow-up visitations. These are not dealt with extensively in this article. Also the low number of aware individuals and consequently under-treatment limited the statistical analysis of data regarding these aspects.

It is therefore not possible to extrapolate our findings to a larger population at country level. However, this study reveals new data about the prevalence, awareness, treatment and control of diabetes and hypercholesterolaemia, and it is the most comprehensive community-based study conducted to date in Angola.

## Future direction

The inclusion of younger participants (15 to 24 years) allows a better representation of the demographic structure of the country and creates a baseline for future surveys. The emphasis for future interventions should be aimed at younger populations in which the prevalence of major risk factors is still low, so as to make a difference in the long term.

In all LMIC, NCDs are the leading cause of death and disability, killing nearly eight million people under 60 years old in 2013.^25^ Over the past decade, the focus of assistance in these countries has primarily addressed maternal and child health and infectious diseases. Without setting these aside, there is an opportunity to use structures that are already in place, to maximise resources. The international community should consider expanding the mandate of current programmes to include outcome-orientated measures for improving general health and lifestyles.

Many of the methods of NCD prevention, management and treatment, which are responsible for the decline in some of these diseases in high-income countries, are inexpensive but are not widely used in LMIC. These methods could be implemented through established global health strategies, such as increased use of low-cost drugs,^35^ and improved access to NCD services for young adults and people with low educational attainment.^36^

## Conclusions

This report reinforces the available data for the main CVD risk factors in Angola and helps to build the basis for further prospective studies, especially among the younger group in this region. We provide the first evidence that hypertension prevalence is rising, together with diabetes, when compared with previous studies in the region.

Despite being a growing economy, Angola’s primary health system may not be currently able to provide an adequate answer to the changing health needs of this population. A gradual shift from infectious diseases to NCDs is underway and this puts additional stress on the reinforcement of primary care intervention in the region.
